# Corrosion Resistance of 2060 Aluminum–Lithium Alloy LBW Welds Filled with Al-5.6Cu Wire

**DOI:** 10.3390/ma11101988

**Published:** 2018-10-15

**Authors:** Fencheng Liu, Xiaoguang Wang, Baosheng Zhou, Chunping Huang, Feiyue Lyu

**Affiliations:** School of Aeronautical Manufacturing and Engineering, Nanchang Hangkong University, Nanchang 330063, China; 160308520412@stu.nchu.edu.cn (X.W.); 160308050312@stu.nchu.edu.cn (B.Z.); cphuang@nchu.edu.cn (C.H.); 17030825J1001@stu.nchu.edu.cn (F.L.)

**Keywords:** 2060 Al Li alloy, laser welding, element segregation, pitting corrosion, intergranular corrosion

## Abstract

Alloy sheets of type 2060 aluminum–lithium were welded by laser beam welding (LBW) filled with ER2319 Al-5.6Cu wire. Microstructural observations showed the uneven distribution of columnar grains, equiaxed grains and equiaxed dendrite grains in the weld. The θ′(Al_2_Cu) phase and other phases precipitated in the weld. The θ′(Al_2_Cu) phase centrally distributed at the grain boundaries. During the immersion corrosion, the pitting corrosion first occurred and then gradually expanded and transformed to intergranular corrosion and exfoliation corrosion. The electrochemical corrosion test showed a higher corrosion tendency of the base metal and heat-affected zone for the lower corrosion potential, but the corrosion current density of the weld was relatively larger. The segregation of Cu, Mg and other elements at the grain boundary aggravated the occurrence of intergranular corrosion.

## 1. Introduction

Aluminum–lithium alloys are finding more and more applications in the aviation industry as researchers aim to achieve weight reduction and strength improvement in skin materials. It is very important for the application of light aluminum alloys. Recently, in order to achieve this purpose, a number of conventional methods have been applied, including microstructure optimization during heat treatment processes, shoot penning, surface engineering, etc. [1,2]. Compared with traditional aluminum alloys, aluminum–lithium alloys have higher specific strength, higher specific stiffness, better heat stability and corrosion resistance, which can significantly improve the properties of aircraft and extend their service life. As a skin material, it will be corroded and destroyed due to the corrosion in the salty atmosphere when the aircraft services coastal areas or marine environments. For some welding structures, the corrosion of welds will be more severe due to the formation of galvanic corrosion behavior of the welds and the base metal with different microstructures and/or different chemical compositions [3].

Type 2060 aluminum–lithium alloy is a kind of the third generation Al–Cu–Li–Mg series alloy and it is strengthened by precipitations formed during aging treatment. It is an ideal aero-material and has been widely used in the aviation industry [4]. During the fabrication of aircraft skins, the riveting of big structures has been replaced by laser beam welding, friction stirring welding and other advanced bonding and welding technologies. Among these welding technologies, laser beam welding (LBW) has obtained special attention for its extensive advantages, including higher energy density, higher welding speed, lower energy input and less influence on base metals. Unfortunately, the weldability and its corrosion behavior of 2060 aluminum–lithium alloy weld are rarely reported and only few researchers have studied the microstructure characteristics or corrosion behavior of aluminum–lithium alloys. Uyime et al. [5] studied the localized corrosion of an AA2198-T851 Al–Li alloy in NaCl solution. The propagation of the corrosion was crystallographic along parallel and intersecting striations aligned parallel to the {111} planes, which is the habit plane of the T_1_ (Al_2_CuLi) phase. The T1 phase is associated with the localized corrosion susceptibility of this alloy. Ma et al. [6] studied the influence of thermomechanical treatments on the localized corrosion susceptibility and propagation mechanisms of AA2099 Al–Li alloy. They found that the severe localized corrosion of the alloy of T8 condition was associated with localized plastic deformation occurring during the pre-age cold working and heterogeneous precipitation of the T_1_ (Al_2_CuLi) phase during subsequent artificial aging. Lin et al. [7] also found that the 2099 Al–Li alloys had different corrosion resistances after different heat treatments. Chen et al. [8] studied the effect of the heat treatment time on the corrosion resistance of Al–Cu–Li–Mg–Mn alloy, and they found that their corrosion resistance became worse when the heat treatment time increased. Other related research [9,10,11,12] has also reported that the quantity and distribution of some Li-, Mg- and Cu-richer precipitates, e.g., T_1_ (Al_2_CuLi) phase, S′ (Al_2_CuMg) phase and T_2_ (Al_6_CuLi_3_) phase, have effects on the corrosion resistance of Al–Li alloys. As a locally rapid melting and rapid solidifying process, the distribution of alloying elements and the microstructure homogeneity of the laser beam welding joints are definitely different from the 2060 Al–Li matrix alloy. This will affect its corrosion resistance in practice correspondingly. Therefore, it is important to figure out the relationship between the microstructure and its corrosion resistance.

In this paper, 2060 Al–Li alloys were welded by fiber laser with ER2319 Al-5.6Cu wire. The microstructure, micro segregation of alloying elements, the phase constitution, and corrosion resistance of the welded joints in 3.5 wt.% NaCl solution were investigated.

## 2. Experimental Methods

The welded materials used in this experiment were 2060-T8 Al–Li alloy sheets with dimensions of 100 mm × 100 mm × 2 mm, and the filling materials were ER2319 Al-5.6Cu wire with a diameter of 1.2 mm. The chemical composition of the 2060-T8 Al–Li alloy sheets and ER2319 wires are listed in Table 1.

The laser beam welding system used in this experiment consisted of an IPG-YLS-6000 model fiber laser ((IPG Photonics, Oxford, MA, USA), KUKA KR16-2W model robot (KUKA Roboter GmbH, Augsburg, Germany), digital-controlled rotary table, revolving platform, gas shielded system and other supplemental devices. The 2016 Al–Li alloy sheets were welded in the form of a butt joint without groove. Before welding, the surface of the sheets was polished and chemically cleaned to remove part of the oxide and oil contamination formed previously. Argon gas was used as the shield gas to avoid the large area oxidation of welds with a flow of 15 L·min^−1^ from both the upper and lower surfaces in the subsequent welding process. The welding direction was parallel to the rolling direction of the sheet. A negative defocusing amount of −2.0 mm was used to achieve the welding of the sheets. The focused laser beam diameter was 0.25 mm, the focal length of the focus mirror was 250 mm, and the focal length of the collimating mirror was 200 mm. The laser power used during the welding was 3.5 kW, with a welding speed of 5 m·min^−1^ and a wire feeding speed of 3.2 m·min^−1^ for all the samples.

After welding, the samples were cut with wire-electrode cutting from every representative region of the joint. The specimens were ground, polished and etched with Keller solution (volume ratio of HF:HCl:HNO_3_:H_2_O = 1:1.5:2.5:95) for the metallographic examination. For microstructure observation, cross-section samples vertical to the welding direction were cut from the joints. An OLMPUS PM-T3 model optical microscope (Olympus Corporation, Tokyo, Japan) and a ZEISS SUPRA 55 model scanning electron microscope (Carl Zeiss Microscopy GmbH, Jena, Germany) were applied for the microstructure observation, and a D8ADVANCE-A25 model X-ray diffractometer (Bruker, Karlsruhe, Germany) was used for the phase constitution analysis in different regions of the joint. To obtain the phase constitution of the weld metal, the weld was cut with wire-electrode cutting into two parts along the central line, and four parts were arranged together to get a thickness of about 8 mm, which was used for the XRD testing. During the X-ray diffraction analysis, Cu Kα radiation (λ = 1.5406 Å) was conducted and the 2*θ* tested ranging from 20° to 80°. The used step size was 0.020498°, and the used step time was 0.38 s/step. Alloying element analysis was also conducted with an EPMA-1720 model scanning probe microscope (Shimadzu Corporation, Tokyo, Japan). This EPMA has the superior specifications unique to the Shimadzu EPMA series, with a high 52.5 degree X-ray take-off angle, and the capacity to accommodate up to five 4-inch, high-sensitivity X-ray spectrometers. The sensitivity to common elements is theoretically 0.01 wt.%. During analysis, an accelerating voltage of 10 kV was used and the distribution of Cu, Si, Mg, Ag and Fe was measured. A transmission electron microscope of the JEM-2010F model (JEOL Ltd., Tokyo, Japan) was used to observe the fine precipitates, and the chemical compositions in these observed areas were examined with an equipped OXFORD INCA EDS analyzer. A disc with a diameter of 3 mm for transmission electron microscopy (TEM) observation was polished to 0.5 mm in thickness after wire-electrode cutting from the central area of the weld. The disc was mechanically thinned down to about 50 μm then electropolished using a double jet with a 30% nitric acid solution in methanol at −20 °C and 15 V. TEM observations were operated at 200 kV.

**Figure 1:**
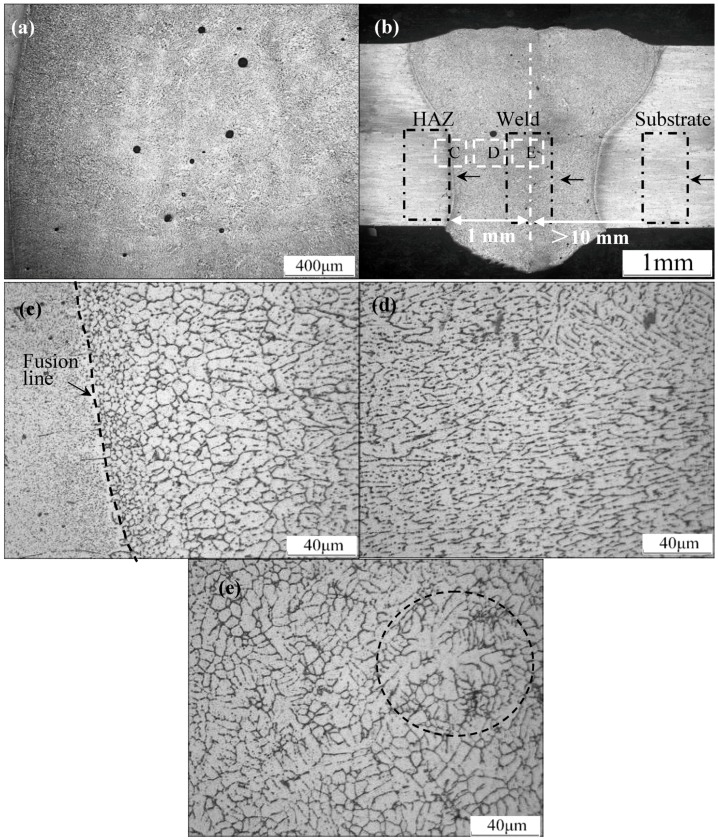
Optical microscopy images of different regions of 2060 Al–Li alloy laser welding joint. (**a**) pores in the top surface of the weld; (**b**) cross-section of the macro morphology of the weld; (**c**) regions of the fusion zone close to the fusion line; (**d**) regions of the fusion zone far from the fusion line; (**e**) central regions of the fusion zone (C, D and E in Figure 1b indicate the position of Figure 1c–e).

To compare the corrosion behavior of different regions of the joint, samples perpendicular to the welding direction were cut and inserted with resin, leaving the cross-section out for corrosion testing. Before corrosion, the surface was polished and cleaned with ethyl alcohol to keep the surface smooth and clean. The corrosion resistance of the different regions of the joints was estimated by static complete immersion tests and electrochemical measurements. Static complete immersion corrosion tests were carried out in 3.5 wt.% NaCl solution at room temperature and the soaking times were 6, 12, 24, 48 and 72 h, respectively. As for electrochemical corrosion testing, samples were cut from the substrate, the heat-affected zone (HAZ) area and the weld metal, respectively. Samples cut from the substrate were used as the substrate testing samples, and the position of the samples cut was far away from the weld, with a distance of more than 10 mm. The samples cut from the weld metal along the weld central line were used as the weld metal testing samples. To get HAZ testing samples, the joints were cut from the fusion line down to the middle height and small blocks were obtained, and the distance of the samples was about 1 mm to the central line of the weld. All the electrochemical corrosion testing samples had a size of 10 mm × 1 mm × 0.5 mm, and the location of each sample is indicated in Figure 1b. The arrows show the testing surface of each sample. The corrosion testing was conducted on the surface of 10 mm × 1 mm. Electrochemical measurements of open circuit potential (OCP) and polarization curve of each sample were conducted to estimate the corrosion resistance. The corrosion cell containing 1000 mL of an electrolyte was combined with a typical three-electrode configuration at room temperature. A saturated calomel electrode (SCE) was used as the reference electrode and a platinum plate was used as a counter electrode (CE). The welded joint specimens were employed as working electrodes (WE). Potentiodynamic polarization tests were swept from −1.2 V to −0.4 V vs. OCPs at a scan rate of 10 mV/s after the tested samples had been immersed in the corrosion solution until steady open circuit potentials (OCPs) were reached. All the corrosion tests were carried out at room temperature (25 °C). After the corrosion tests, the corrosion morphologies were observed with an optical microscope and a scanning electron microscope.

## 3. Results and Discussion

### 3.1. Macro- and Micro-Structures of the Weld

As with other aluminum alloys [13,14,15], pores are easily formed during the laser beam welding of the 2060 Al–Li alloy filled with ER2319 wires, more pores are observed on the top of the weld and the size is large, compared to the pores on the low part of the weld, as shown in Figure 1a,b. The size of the larger pores was about 60 μm and the small pores were only about 25 μm. It was hard to determine the kindness of the pores according to their distribution location and morphologies; however, according to the analysis of Song et al. [16], the precipitation of supersaturated H from liquid metal during the solidification of the molten pool will result in the formation of metallurgical pores with a different size, shape and amount. A part of the moisture, which may have come from the non-pre-heated wire and/or the sheet surface and could not be removed by shielding gas, was easily adsorbed into the liquid metal during welding process because the Al–Li alloy has high moisture absorption performance. This will lead to the formation of compounds containing oxygen and/or hydrogen, e.g., LiOH, Mg(OH)_2_, Li_2_O and MgO. Meanwhile, hydrogen is easy to solute into the molten pool at a high concentration and will remain in the weld in the form of pores, since there is not enough time allowing it to escape from the weld. The evaporation of low-boiling point elements such as Li and Mg also has an influence on the formation of pores in the weld, lower Li and Mg element contents can effectively reduce the pores in the weld [17,18].

The microstructures of different regions in the cross-section of the weld are shown in Figure 1c–e. It can be seen that distinct differences in the morphology and grain size of different regions existed in the weld. There was a fine equiaxed grain region along the fusion line in the weld zone with a width of about 20 μm on both sides of the weld. Next to the fine equiaxed grain region was a columnar grain region with a sub-structure of cellular crystals in the weld. Coarse equiaxed grains with fully developed equiaxed dendrites formed in the center of the weld, because the cooling rate was slow here, and secondary dendrite arms were well-developed in this region, as shown in Figure 1e. The fine grain structure reflects that the cooling rate and temperature gradient of the liquid metal in this region were high during solidification. Closer to the center of the weld, the cooling rate and temperature gradient were lower. Coarse grains formed in the corresponding regions, which changed from fine equiaxed grains to columnar grains, consisting of cellular and dendrite, and equiaxed dendrite grain with a large size distributed in the center.

### 3.2. Distribution of Alloying Elements and Secondary Phase Constitution

**Figure 2:**
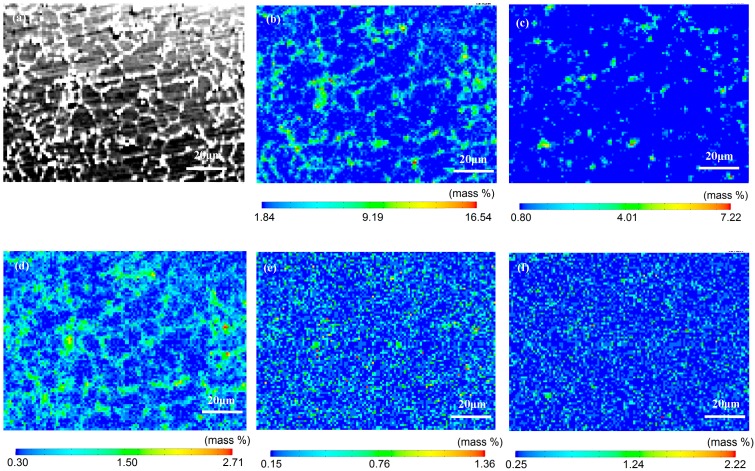
Electro-Probe Microanalyzer (EPMA) analysis of the chemical compositions of the weld metal in the 2060 Al–Li alloy laser welding joint. (**a**) microstructure of the fusion zone; (**b**) distribution of Cu; (**c**) distribution of Si; (**d**) distribution of Mg; (**e**) distribution of Ag; (**f**) distribution of Fe.

Electron microprobe analysis was used to measure the distribution of alloying elements in the weld, and the results are shown in Figure 2. As shown in Figure 2a, some white precipitates were distributed at the grain boundaries. Figure 2b–d suggests that some alloying elements, for example Cu, Mg and Si, were segregated in the grain boundaries, while Figure 2e,f present the even distribution of Ag and Fe in the weld. As known from the chemical composition of the ER2319 Al-5.6Cu wires in Table 1, the contents of Cu, Si and Fe of the weld will increase due to the use of a filling material, and this is beneficial to the strengthening of the weld by accelerating the precipitation of the θ′(Al_2_Cu) phase and other phases during post-heat treatment [19]. However, the addition of Cu and Si also promoted the precipitation of secondary phases at the grain boundaries.

**Figure 3:**
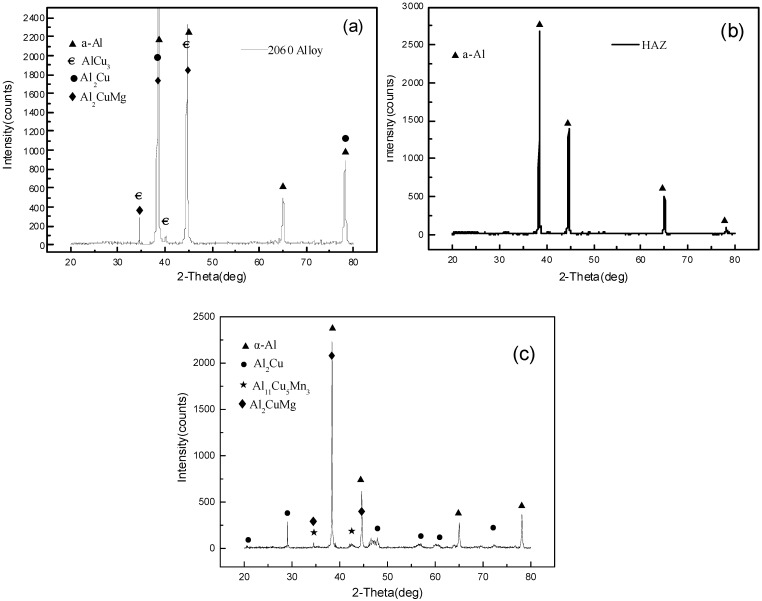
X-ray diffraction spectrum of the 2060 Al–Li alloy laser welding joint. (**a**) 2060 Al–Li alloy base metal; (**b**) HAZ; (**c**) weld metal.

**Figure 4:**
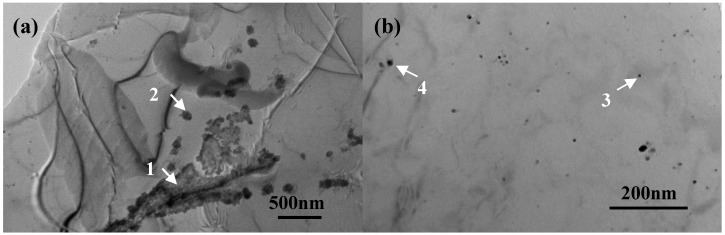
TEM images of the fusion zone of the 2060 Al–Li alloy laser welding metal. (**a**,**b**) are microstructure images of the grain boundaries and inner grains.

The phase constitution of the joint was analyzed by X-ray diffraction analysis and the results are shown in Figure 3. The strong diffraction peaks of the θ′(Al_2_Cu) phase indicates a large amount of these precipitates formed in the weld. The results reveal that α-Al was the main phase of the whole joint, and the θ′(Al_2_Cu) phase, S′(Al_2_CuMg) phase and other phases also existed in the 2060 Al–Li alloy base metal. Fewer phases were found in the HAZ area, as shown in Figure 3b, which shows the solution effect to this area by heat input during laser welding. For the weld metal, the α-Al, θ′(Al_2_Cu) phase and little S′(Al_2_CuMg) phase were detected, as shown in Figure 3c. It can be deduced that the secondary phase distributed at the grain boundaries was the primary θ′(Al_2_Cu) phase precipitated directly from the molten liquid, because its amount was the largest indicated by XRD analysis. It is well known that the θ′(Al_2_Cu) phase is also the primary strengthening phase for 2060 alloy and other Al–Li alloys [20,21,22]. Its uniform precipitation in base metal can effectively strengthen the alloy after solution and aging treatment. However, in the condition of laser beam welding, the rapid cooling of the weld could prohibit the complete precipitation of the θ′(Al_2_Cu) phase, if not enough time is allowed for its precipitation from the α-Al base metal. As there is a too strong overlap with θ′(Al_2_Cu) diffraction peaks, it is difficult to determine whether or not these phases formed in the weld. Transmission electron microscopy was used to observe the morphology of the secondary phases in the weld, and some images are shown in Figure 4.

Figure 4 exhibits the TEM images of grain boundaries and the inner grains of the weld. It can be seen that there were more secondary phases precipitates in the grain boundary area than in the inner grain area in Figure 4b, as marked by the arrow in Figure 4a. The precipitation of the secondary phase needs enough time for their nucleation and growth. However, in welded conditions, as observed in this experiment, the number of secondary phases was low and their size was small, because there was not enough time for nucleation and growth. The volume fraction of large θ′(Al_2_Cu) at the grain boundaries was measured by Image Pro Plus software in the weld metal area, and the volume fraction was about 10.8 vol.%. However, as shown in the TEM images in Figure 4, less precipitates formed in the inner grain area, and its volume fraction was less than 0.9 vol.%. This value was much lower than that in normal 2060 Al–Li alloy, as shown in reference [22]. The EDS measurements of these precipitations are listed in Table 2. The secondary particles at the grain boundaries were large and contained more Cu, but those in the inner grain area were small and contained less Cu. As the distribution of Cu has an important influence on the corrosion resistance, and the segregation of Cu at grain boundaries will increase its corrosion tendency, thus it can be indicated that the corrosion resistance of the inner grain area was much better than the grain boundary area. Even though lesser precipitation will lead to lower strength, the homogeneous structure will result in higher resistance to corrosion.

### 3.3. Immersion Corrosion of the Weld

**Figure 5:**
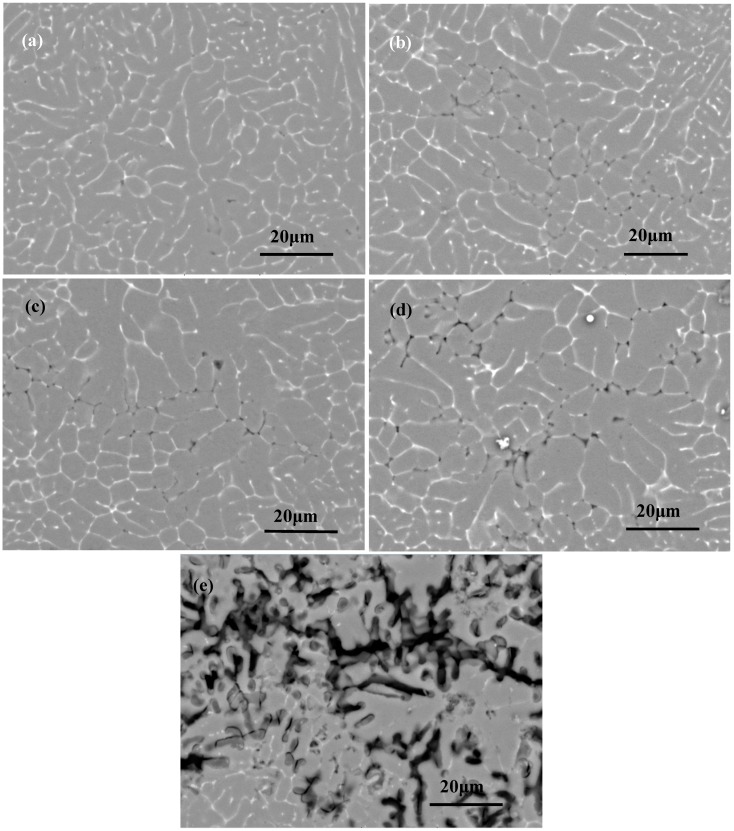
Surface morphologies of the 2060 Al–Li alloy laser welding joint after immersion corrosion in 3.5% NaCl solution. (**a**) 6 h; (**b**) 12 h; (**c**) 24 h; (**d**) 48 h; (**e**) 72 h.

Figure 5 shows the surface morphologies of the 2060 Al–Li alloy laser welding joint after immersion corrosion in 3.5% NaCl solution for different times. It can be seen that after immersion for 6 h, only few locations showed pitting corrosion at the grain boundaries. With the increasing immersion time, the pitting corrosion became more serious, as shown in Figure 5b, and then the size of the pits enlarged, as shown in Figure 5c. After immersion for 48 h, the pitting corrosion gradually expanded and changed to intergranular corrosion, as shown in Figure 5d. A longer time immersed in the NaCl solution caused the serious corrosion of boundaries, as shown in Figure 5e.

**Figure 6:**
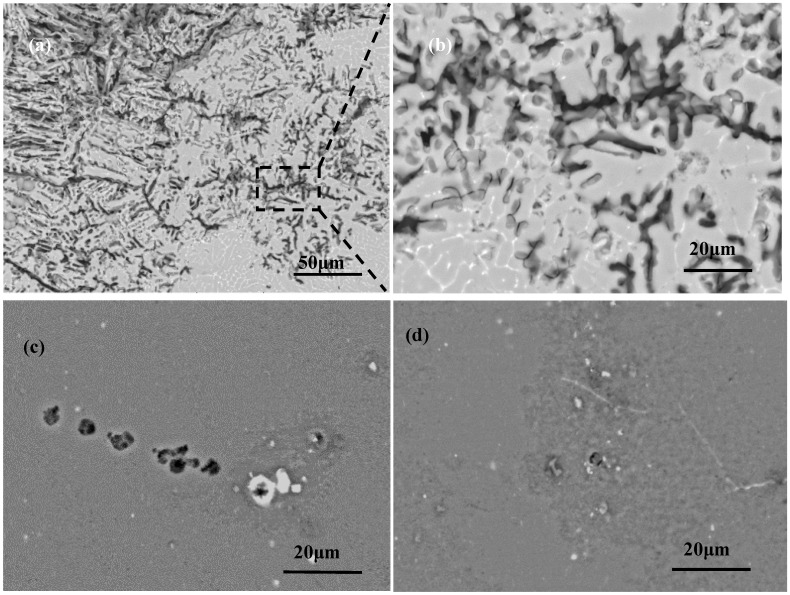
Surface morphologies of local areas of the 2060 Al–Li alloy laser welding joint after immersion corrosion in 3.5% NaCl solution for 72 h. (**a**) weld metal; (**b**) partially enlarged detail of the weld metal; (**c**) base metal; (**d**) heat-affected zone.

Figure 6 demonstrates the surface morphologies of local areas of the 2060 Al–Li alloy laser welding joint after immersion corrosion in 3.5% NaCl solution for 72 h. As shown in Figure 6a, serious corrosion occurred in the weld metal area. Figure 6b shows that intergranular corrosion occurred in the interdendritic area. Compared with the weld metal, the surface morphology of the 2060 base metal and the heat-affected zone (HAZ) were smoother without any corrosion pits after immersion corrosion in 3.5% NaCl solution for 72 h (as shown in Figure 6), and the corrosion occurred at the grain boundaries of the base metal and the heat-affected zone (HAZ). It can be deduced that the occurrence of pitting in the base metal and the HAZ were due to the existence of secondary phase particles. As shown in Figure 6c, the existence of large size secondary phase particles and the microsegregation of alloying elements resulted from the precipitation of secondary phase particles in these areas, and caused the occurrence of pitting. In the HAZ region, large size secondary phase particles dissolved back into the matrix metal and disappeared due to the solution effect of the welding thermal cycles, as seen in Figure 6d. Pitting assembled around small secondary phase particles. Figure 6 clearly indicated that the corrosion resistance in different regions of the joint were different, and that was why the HAZ area presented the best corrosion resistance and the weld revealed the worst wear after longer immersion corrosion in 3.5% NaCl solution. Therefore, the microstructural homogeneity has an important influence on the corrosion behavior of the weld.

### 3.4. Electrochemical Corrosion of the Weld

**Figure 7:**
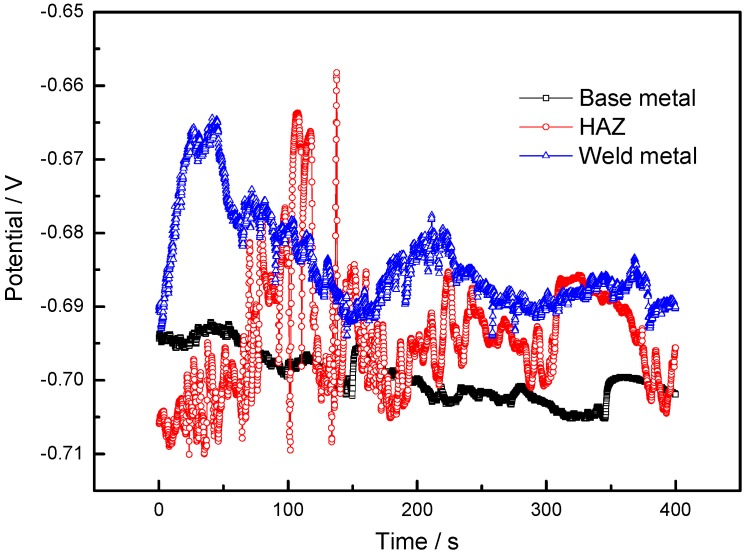
Corrosion potentials of the base metal, heat-affected zone and weld metal in 3.5% NaCl solution.

Open circuit potential is the potential difference of the working electrode and the reference electrode when no voltage is applied to the cycle, which is also the potential value when the current density is zero on the surface of the electrodes. The OCP is a reliable parameter that indicates the tendency of the corrosion process, and the measurement of OCP is generally performed to study the chemical stability of a testing sample. As is known, the stabilized electrode potential reflects the corrosion probability, and the more negative the electrode potential, the greater the probability that the electrode will be corroded. Figure 7 shows the variations of the corrosion potential of the 2060 base metal, HAZ and weld metal during the OCP tests. The OCP as a function of the immersion time for different regions of the joint are provided in Figure 6. It was found that the stabilized electrode potentials of the 2060 base metal and HAZ were −0.7019 V and −0.6956 V in Figure 7, respectively. These two potentials were more negative than that of the weld metal, which was −0.6902 V. Thus, the 2060 base metal and HAZ had a higher corrosion tendency and were more likely to be corroded. This confirms that the base metal has higher chemical stability than the weld and HAZ in the 3.5% NaCl solution.

It should be pointed out that the corrosion tendency can only provide the possibility of corrosion, and whether it will occur is controlled by many other factors, e.g., the microstructure of the electrode surface. For this reason, the electrochemical corrosion tests of the potentiodynamic polarization curve were used to analyze their corrosion mechanism.

**Figure 8:**
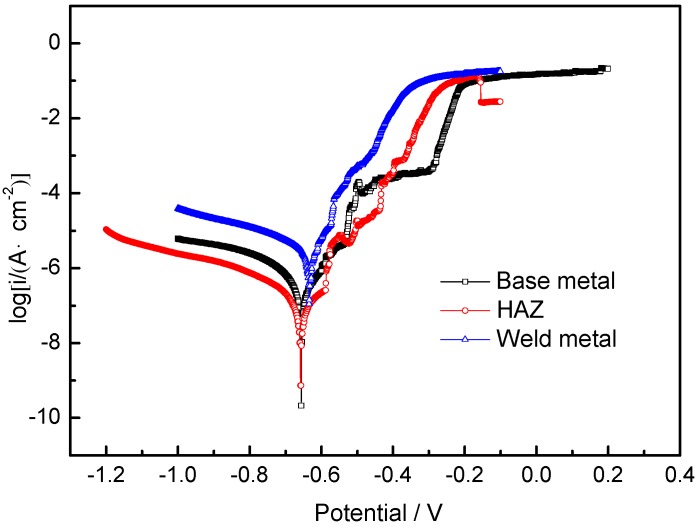
Polarization curves of different regions of the weld in 3.5% NaCl solution.

Figure 8 shows the polarization curves of base metal, heat-affected zone and weld metal in 3.5% NaCl solution. Table 3 suggests the corrosion potential E_corr_ and corrosion current density I_corr_ for each sample. Anodic active dissolution occurred in all these three areas. It was found that the corrosion potentials of the 2060 base metal, HAZ and the weld metal were similar, and the conclusion can be drawn that the corrosion tendencies in these three areas might be similar as those deduced from similar corrosion potentials [23,24,25].

As listed in Table 3, the corrosion current density of the HAZ was the lowest, at 5.880 × 10^−8^ A/cm^−2^, indicating that the corrosion rate of this region was the lowest. Meanwhile, the corrosion current density of the weld metal, which was 3.548 × 10^−6^ A/cm^−2^, was higher than that of the base metal (5.877 × 10^−8^ A/cm^−2^). The analysis showed that the corrosion current density increased due to the high content of Cu and Mg in the filler material. The corrosion current density of the 2060 base metal (5.877 × 10^−7^ A/cm^−2^) was higher than that of the HAZ (5.880 × 10^−8^ A/cm^−2^), and this means that the corrosion rate of the HAZ was lower. The corrosion rate of the weld metal was the highest, the 2060 base metal was in second place, and the HAZ was the lowest. The corrosion rates of different regions were considered to be affected by the homogeneity of the microstructure and the alloying element distribution. As shown in Figure 1 and Figure 2, more secondary phase precipitates in the grain boundary area and the degree of Cu, Mg and Si segregation was relatively large in this area. The corrosion current density of the weld metal was higher than the other two areas, and the corrosion resistance of the weld metal was the worst. Compared with the base metal, the secondary particles at the grain boundaries of the HAZ were dissolved back into the matrix metal, thus the HAZ region had a more homogeneous microstructure and better corrosion resistance.

## 4. Conclusions

1. The weld mainly contains α(Al) phase columnar grains. Fine equiaxed grains exist adjacent to the fusion line and equiaxed dendrite grains exist in the center of the weld. Besides the α(Al) matrix metal, secondary precipitates, e.g., θ′(Al_2_Cu) phase and very small amounts of inclusions, such as Al_11_Cu_5_Mn_3_ and Al_7_Cu_2_Fe, formed in the weld.
2. At the beginning of immersion corrosion in 3.5% NaCl solution, corrosive pitting was the main corrosion. With the increase of the soaking time, the pitting corrosion transformed into intergranular corrosion for the connection of corrosion pits. This was related to the segregation of Cu and Mg in local areas of the weld.
3. Electrochemical corrosion tests show that the corrosion resistance of the 2060 base metal and the HAZ regions were better than that of the weld metal. Due to the alloying element segregation and the existence of large-size secondary phases, the corrosion resistance of the weld metal was the worst.

## Figures and Tables

**Table 1:** Chemical composition of the 2060 Al–Li alloy and ER2319 wire (mass fraction. %).

Alloy	Cu	Li	Mg	Zn	Mn	Ag	Zr	Si	Fe	Al
2060	3.80	0.75	0.85	0.42	0.30	0.30	0.11	0.05	0.10	Bal.
ER2319	5.60	/	0.20	0.10	0.30	--	--	0.20	0.30	Bal.

**Table 2:** EDS analysis of the chemical composition of the precipitation in positions marked out in Figure 4.

	Element	Al	Cu	Ag	Mn	Fe
1	wt.%	21.9	71.7	5.8	/	0.6
	at.%	40.5	56.3	2.7	/	0.5
2	wt.%	20.2	79.8	/	/	/
	at.%	37.4	62.6	/	/	/
3	wt.%	77.0	5.7	16.8	0.4	0.5
	at.%	91.6	2.9	5.0	0.2	0.3
4	wt.%	92.1	4.4	2.8	0.7	/
	at.%	97.0	2.0	0.8	0.4	/

**Table 3:** Corrosion potential Ecor and corrosion current density Icor of different regions of the weld during electrochemical corrosion in in 3.5% NaCl solution.

Regions	E_corr_ (V)	I_corr_ (A/cm^−2^)
2060 base metal	−0.656	5.877 × 10^−7^
HAZ	−0.658	5.880 × 10^−8^
Weld metal	−0.634	3.548 × 10^−6^
